# Harnessing Exhaled Breath for Lung Cancer Early Detection—Results From the ExPeL Study

**DOI:** 10.1111/cts.70687

**Published:** 2026-07-29

**Authors:** Danita Kasi Patel, Leon D'Cruz, Waqar Ahmed, Anoop Chauhan, Nawar Diar Bakerly, Seamus Grundy, Drupad K. Trivedi, Sean Knight

**Affiliations:** ^1^ Manchester Institute of Biotechnology (MIB), Department of Chemistry University of Manchester Manchester UK; ^2^ School of Medicine, Pharmacy and Biomedical Sciences, Faculty of Science and Health University of Portsmouth Portsmouth UK; ^3^ Research and Innovation Department Portsmouth Hospitals University NHS Trust Portsmouth UK; ^4^ Division of Immunology, Immunity to Infection and Respiratory Medicine; School of Biological Sciences; Faculty of Biology, Medicine and Health University of Manchester Manchester UK; ^5^ Respiratory Department, Salford Care Organisation Northern Care Alliance Foundation Trust Salford UK; ^6^ Lydia Becker Institute of Immunology and Inflammation University of Manchester Manchester UK

## Abstract

Scalable, non‐invasive tools are critically needed to improve early lung cancer detection and optimize primary care referral pathways. We evaluated Inflammacheck, a point‐of‐care device utilizing exhaled breath condensate (EBC) H_2_O_2_ and physiological parameters with machine learning for non‐invasive lung cancer detection in a real‐world screening population. Exhaled Hydrogen Peroxide for Early Lung Cancer Detection (ExPeL) study participants, from the UK Targeted Lung Health Check (TLHC) programme, included individuals with suspected lung cancer and low‐risk ever‐smoker controls. EBC was collected via Inflammacheck, measuring H_2_O_2_, end‐tidal CO_2_, humidity, temperature, and exhalation flow rate. Multivariate analyses (PCA, LDA and Mahalanobis distance) assessed intrinsic group separation. SMOTE‐balanced data trained supervised machine learning models (stacked and voting ensembles), which were then evaluated on held‐out test sets. In parallel, untargeted LC–MS metabolomics was performed to identify discriminatory molecular features. Analyzing 34 participants with valid EBC data, 83% of cancer cases were early‐stage (I–II), reflecting a screening population. Multivariate analysis clearly separated lung cancer and controls across PCA, LDA, and Mahalanobis mapping. The voting ensemble model achieved: Accuracy 85.7%, Sensitivity 80%, Specificity 100%, Precision (PPV) 100%, ROC–AUC 0.90 and MCC 0.73. Crucially, no false positives were identified. EBC variables revealed greater dispersion in cancer patients, reflecting physiological heterogeneity missed by univariate analysis. Untargeted metabolomics identified 2132 features, with four key metabolites yielding an AUC of 0.969 for cancer discrimination. Inflammacheck effectively distinguishes early‐stage lung cancer via a rapid, non‐invasive breath test, findings which are highly relevant for primary care and screening triage, where non‐specific symptoms and low prevalence pose challenges.

## Study Highlights

- What Is the Current Knowledge on This Topic?
  ○ Hydrogen peroxide is produced in the tumor microenvironment and can be detected in exhaled breath. Studies in late stage cancer show that it can distinguish lung cancer from other respiratory disorders when this measurement is incorporated into a machine learning algorithm with other breath parameters.
- What Question Does This Study Address?
  ○ This study (ExPeL) explores whether exhaled hydrogen peroxide and other breath parameters, measured in a new device called inflammacheck, can distinguish early‐stage lung cancers from ever smokers eligible for the UK Targeted Lung Health Check program. It also included an independent analysis to look for new exhaled molecules associated with lung cancer that could be added to inflammacheck in the future to improve performance.
- What Does This Study Add to Our Knowledge?
  ○ The data collected by infalmmacheck can be used to distinguish early lung cancer patients from controls with high accuracy.
- How Might This Change Clinical Pharmacology or Translational Science
  ○ Inflammacheck has potential to be used in the UK Targeted Lung Health Check program to improve the efficiency of screening by reducing the number of CTs needed.

## 1 Introduction

For cost efficiency, most lung cancer screening programs use a pre‐screen clinical score to enrich a high‐risk population. In the UK, this means that only 40% of people with lung cancer would have been eligible for CT screening prior to their diagnosis [1]. New tests are needed to focus provision of CT scans more effectively. Expired air contains a rich source of molecules that can be sampled non‐invasively to discover new tests. Rapid cooling of breath leads to exhaled breath condensate (EBC), which contains sediments of aerosolised particles. EBC analysis has previously identified metabolic compounds associated with late‐stage lung cancer [2], but it is not clear whether these extrapolate to early stage disease [3]. Reactive oxygen species (ROS) are promising biomarkers as they are readily produced in the tumor microenvironment by both cancer and immune cells [4]. Hydrogen peroxide in EBC was significantly elevated in patients with non‐small cell lung cancer compared to controls in two studies [5, 6]. However, both studies excluded patients with chronic respiratory disorders, which independently increase ROS in EBC as reviewed previously [7].

Inflammacheck is a handheld device that measures hydrogen peroxide via an enzymatic reaction within a sensor, providing point‐of‐care, accurate measurements. It also measures peak end‐tidal carbon dioxide, peak breath humidity, peak breath temperature (°C), and mean exhalation flow rate. Given that exhaled hydrogen is also increased in chronic lung disease, inclusion of breath parameters is important to determine whether observed readings are proportional to the degree of physiological impairment in breathing. Thus, lung cancer can be distinguished by hydrogen peroxide levels out of proportion to underlying chronic lung disease burden. A machine learning algorithm was developed to this effect in the VICTORY study [8].

In this study (ExPeL), we extend the findings from VICTORY, testing the diagnostic accuracy of Inflammacheck in populations relevant for lung cancer screening. In parallel, we have performed an unbiased screen of EBC for novel molecular candidates, identifying new metabolites associated with lung cancer that will be incorporated into Inflammacheck in the future.

## 2 Methods

### 2.1 Study Design and Participant Recruitment

The Harnessing Exhaled Hydrogen Peroxide for Early Lung Cancer Detection (ExPeL) study received ethical approval from the West Midlands NHS Research Ethics Committee (IRAS: 336691; REC: 24/WM/0028; ISRCTN: 81020233). All participants provided written informed consent.

Participants aged ≥ 16 years were recruited from the Greater Manchester Lung Cancer Screening service. **Cases** comprised individuals with radiologically suspected lung cancer referred for CT‐guided biopsy following computed tomography (CT) imaging undertaken within the Targeted Lung Health Check (TLHC) programme, for respiratory or cancer‐related symptoms (‘symptomatic’), or for non‐respiratory indications (‘incidental’). TLHC is part of NHS England's targeted screening initiative for ever‐smokers, designed to improve early lung cancer detection in high‐risk populations [9].

**Controls** were defined as ever‐smokers assessed through the TLHC pathway who underwent low‐dose CT imaging and were not diagnosed with lung cancer following guideline‐directed radiological assessment and follow‐up. This included individuals with pulmonary nodules classified as low risk according to national nodule management criteria, and one participant with histologically confirmed benign disease. We reiterate that the control group represents screen‐negative, low‐risk TLHC participants, not lifelong benign controls. Detailed justification of control classification is provided in the online‐Supplementary file.

### 2.2 Exhaled Breath Condensate Collection

Exhaled breath condensate (EBC) was collected using the Inflammacheck device. Participants breathed tidally through a disposable mouthpiece while wearing a nose clip for up to 6 min; sampling terminated automatically once sufficient condensate was obtained. Five parameters were recorded: hydrogen peroxide concentration (μM), peak end‐tidal carbon dioxide (%), peak humidity (%), peak breath temperature (°C), and mean exhalation flow rate (L/min).

A subset of participants also provided an additional EBC sample using the Coronacheck device to enable larger‐volume collection for downstream metabolomic analysis. EBC measurements were obtained for research purposes only and did not inform clinical management.

### 2.3 Data Quality Control and Sample Exclusion

Following quality review, a sensor equilibration issue affecting a subset of Inflammacheck devices was identified, resulting in unreliable humidity‐dependent measurements. The protocol was amended and additional participants were recruited. Of 56 participants undergoing Inflammacheck testing, 15 datasets were excluded due to sensor artifact and 7 due to insufficient sample acquisition, leaving 34 valid datasets for analysis.

### 2.4 Multivariate (PCA, LDA and Mahalanobis Distance Analysis) Analysis

All EBC variables were standardized to zero mean and unit variance prior to multivariate analysis. Principal component analysis (PCA) was performed using singular value decomposition to obtain orthogonal linear combinations of the original variables that maximize explained variance. The first two principal components were used for visualization of global variance structure. Convex hulls were applied to illustrate within‐group dispersion; these were descriptive and not inferential.

Linear discriminant analysis (LDA) was subsequently performed to identify the linear combination of features that maximized separation between cancer and non‐cancer groups. For two classes, LDA produces a single discriminant axis defined by maximizing the ratio of between‐class scatter to within‐class scatter. Projection onto this axis was used to visualize supervised class separation.

Mahalanobis distances were calculated, scripted in Python, using pooled within‐class covariance. Class‐specific covariance matrices were computed and combined into a pooled estimate:

$$S_{\text{pooled}}=\frac{\left(n_{\mathrm{NC}}-1\right)S_{\mathrm{NC}}+\left(n_{C}-1\right)S_{C}}{n_{\mathrm{NC}}+n_{C}-2}$$

The inverse pooled covariance matrix was used to compute covariance‐adjusted distances of each observation to both class centroids:

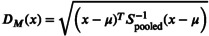




Distances were visualized as a heatmap to illustrate relative proximity to each class centroid.

### 2.5 Machine Learning Analysis

To address class imbalance inherent to real‐world screening cohorts, the Synthetic Minority Oversampling Technique (SMOTE; generation of synthetic minority‐class samples in feature space) was applied to the training data only [10]. Machine learning models were optimized using grid‐search hyperparameter tuning with five‐fold cross‐validation, executed on a high‐performance computing cluster (SCIAMA, University of Portsmouth).

Two ensemble classifiers were evaluated: a **stacked ensemble** (Random Forest and XGBoost base learners with logistic regression meta‐learner) and a **soft voting ensemble** (Random Forest, XGBoost, logistic regression). Models were trained on SMOTE‐balanced data and evaluated on held‐out test sets. Full methodological details are provided in the online‐Supporting Information.

### 2.6 Metabolomic Analysis

This analysis applies to data presented in Figure 3. EBC metabolites were extracted using methanol and analyzed by liquid chromatography–mass spectrometry (LC–MS). Compound identification employed GNPS spectral matching and SIRIUS‐based in silico fragmentation. All annotations were classified as Metabolomics Standards Initiative (MSI) level 3. Data were normalized to sample weight, log‐transformed, Pareto‐scaled, and analyzed using sparse partial least squares discriminant analysis (sPLS‐DA) in MetaboAnalyst 5.0. Feature importance was assessed using VIP scores and statistical testing, followed by pathway enrichment analysis.

## 3 Results

### 3.1 Study Population and Clinical Characteristics

**FIGURE 1:**
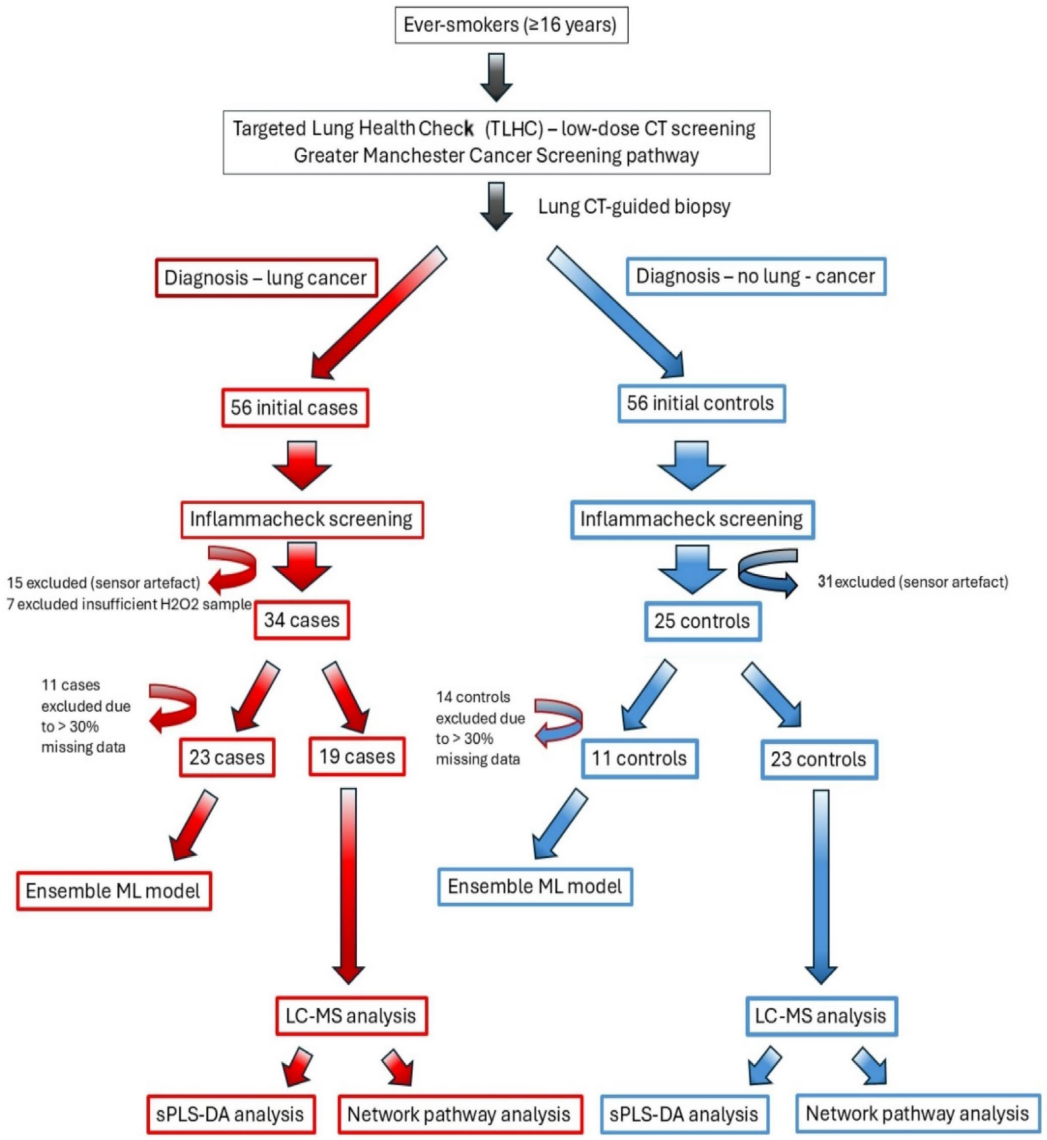
Schematic diagram showing the recruitment of participants into the ExPeL study. Participants; cases (in red) and controls (in blue), were recruited from ever‐smokers who were invited to participate in the national screening for lung‐cancers via the Targetted Lung Health Check programme, offering low‐dose CT screening for early‐detection of lung‐cancers. ExPeL included two independent analyses: An Ensembl ML model using data from the inflammacheck device and an unbiased molecular screen by liquid chromatography mass spec from EBC samples collected from the coronacheck device. The patient numbers are noted in the figure and annotated with all the exclusions. Onward management of patients is guided primary by the NHS‐England's TLHC protocol, the British Thoracic Society (BTS) pulmonary nodule guidelines and the local lung cancer Multi‐disciplinary team (MDT) processes. Patients referred for onward management of suspected lung‐cancers are referred for CT‐guided biopsy at secondary or tertiary referral centres. A patient is placed on the cancer‐pathway for management if the histology confirms a malignancy, of there is a positive cytology sample (EBUS‐sample). These participants are the cases (in red) who eventually are recruited (following written informed consent) for Inflammacheck screening or for providing a sample to be analyzed by LC–MS. Participants who have a nodule size < 5 mm or < 80 mm^3^ are discharged from the suspected‐cancer‐pathway with no need for subsequent‐follow‐up (as per BTS guidelines). Patients from this subset were approached to participate in Inflammacheck screening and to provide samples for LC–MS analysis following written informed consent.

Staggered recruitment of participants (cases and controls) following written informed consent to the ExPeL study. The recruitment pathway is summarized in Figure 1, among lung‐cancer cases, 83% had early‐stage disease (Stage I–II). Mean age was similar between cases and controls (69.9 vs. 68.4 years), with a slightly higher proportion of males among controls (64% males vs. 36% females). Subsets used for Inflammacheck machine‐learning analysis and LC–MS metabolomics were broadly representative of the full cohort in terms of age and sex, supporting internal consistency across analyses (Table 1).

**TABLE 1:** Participant demographics and clinical characteristics across overall cohort and analytical subsets. Data are presented as *n* (%). The table summarizes age, sex, comorbidities, referral route, smoking status, lung cancer stage, and histology for all participants, as well as for the subsets used in Inflammacheck exhaled breath condensate (EBC) machine‐learning analysis and LC–MS metabolomics exploratory analysis. Comorbidities include ischemic heart disease (IHD), hypertension (HTN), diabetes, chronic obstructive pulmonary disease (COPD), asthma, and prior non‐lung cancers. Lung cancer histologies include lung adenocarcinoma (LUAD), squamous cell carcinoma (LUSC), small cell lung cancer (SCLC), carcinoid, neuroendocrine, mixed, and non‐small cell lung cancer not otherwise specified (NSCLC NOS).

	All participants		Inflammacheck EBC‐machine‐learning analysis		EBC LC–MS exploratory analysis	
	Cases	Controls	Cases	Controls	Cases	Controls
Participants	34	25	23	11	19	23
Mean age (years)	69.9	68.4	69.4	69.7	70.46	68
Male (%)	48	64	48	56	47	61
Female (%)	52	36	52	44	53	39
*Comorbidities*						
IHD	5 (16.1)	1 (4)	4 (17.4)	1 (9.1)	5 (26.3)	0 (0)
HTN	7 (22.6)	2 (8)	6 (26.1)	2 (18.2)	5 (26.3)	2 (8.7)
Diabetes	12 (38.7)	2 (8)	8 (34.8)	2 (18.2)	7 (36.8)	1 (4.3)
COPD	6 (19.4)	4 (16)	4 (17.4)	0 (0)	5 (26.3)	3 (13)
Asthma	3 (9.7)	3 (12)	2 (8.7)	1 (9.1)	3 (15.8)	3 (13)
Previous cancer	1 (3.2)	6 (24)	0 (0)	3 (27.3)	1 (5.3)	6 (26)
*Referral route*						
Incidental	5 (16.1)		3 (13)		2 (10.5)	
Symptomatic	10 (32.3)		8 (34.8)		8 (42.1)	
Lung cancer screening	16 (51.6)		12 (52.2)		9 (47.4)	
*Smoking status*						
Current	8 (25.8)	5 (20)	7 (30.4)	0 (0)	5 (26.3)	5 (21.7)
Former	19 (61.3)	20 (80)	13 (56.6)	11 (100)	12 (63.2)	18 (78.3)
Never	4 (12.9)	0 (0)	3 (13.0)	0 (0)	2 (10.5)	0 (0)
*Cancer stage*						
1	19 (61.3)		15 (65.2)		11 (57.9)	
2	7 (22.6)		5 (21.7)		5 (26.3)	
3	2 (6.5)		1 (4.4)		2 (10.5)	
4	2 (6.5)		2 (8.7)		1 (5.2)	
*Cancer histology*						
LUAD	15 (48.4)		9 (39.1)		9 (47.4)	
LUSC	6 (19.4)		5 (21.7)		5 (26.3)	
SCLC	2 (6.5)		3 (13)		2 (10.5)	
Carcinoid	2 (6.5)		2 (8.7)		1 (5.3)	
Neuroendocrine	1 (3.2)		1 (4.4)		0 (0)	
Mixed	1 (3.2)		1 (4.4)		1 (5.3)	
NSCLC NOS	3 (9.6)		2 (17.4)		1 (5.3)	
Unknown	1 (3.2)		0		0	

Comorbidity profiles differed between cases vs. control groups. Diabetes (34%–39% vs. 4%–18%) and ischaemic heart disease (16%–26% vs. 0%–9%) were more prevalent among cases, while prior non‐lung malignancies were more common in controls (24%–27%). Chronic respiratory disease prevalence (COPD and asthma) was comparable. Smoking history was similar across groups, with the majority being former smokers.

Most lung cancers were detected through screening pathways (47%–52%), with the remainder identified incidentally or via symptomatic presentation. Early‐stage disease predominated (58%–65% Stage I), and adenocarcinoma was the most common histological subtype (39%–48%), followed by squamous cell carcinoma and small cell lung cancer. These distributions were preserved across analytical subsets.

### 3.2 Exploratory Multivariate Separation of Cancer and TLHC‐Excluded Participants

**FIGURE 2:**
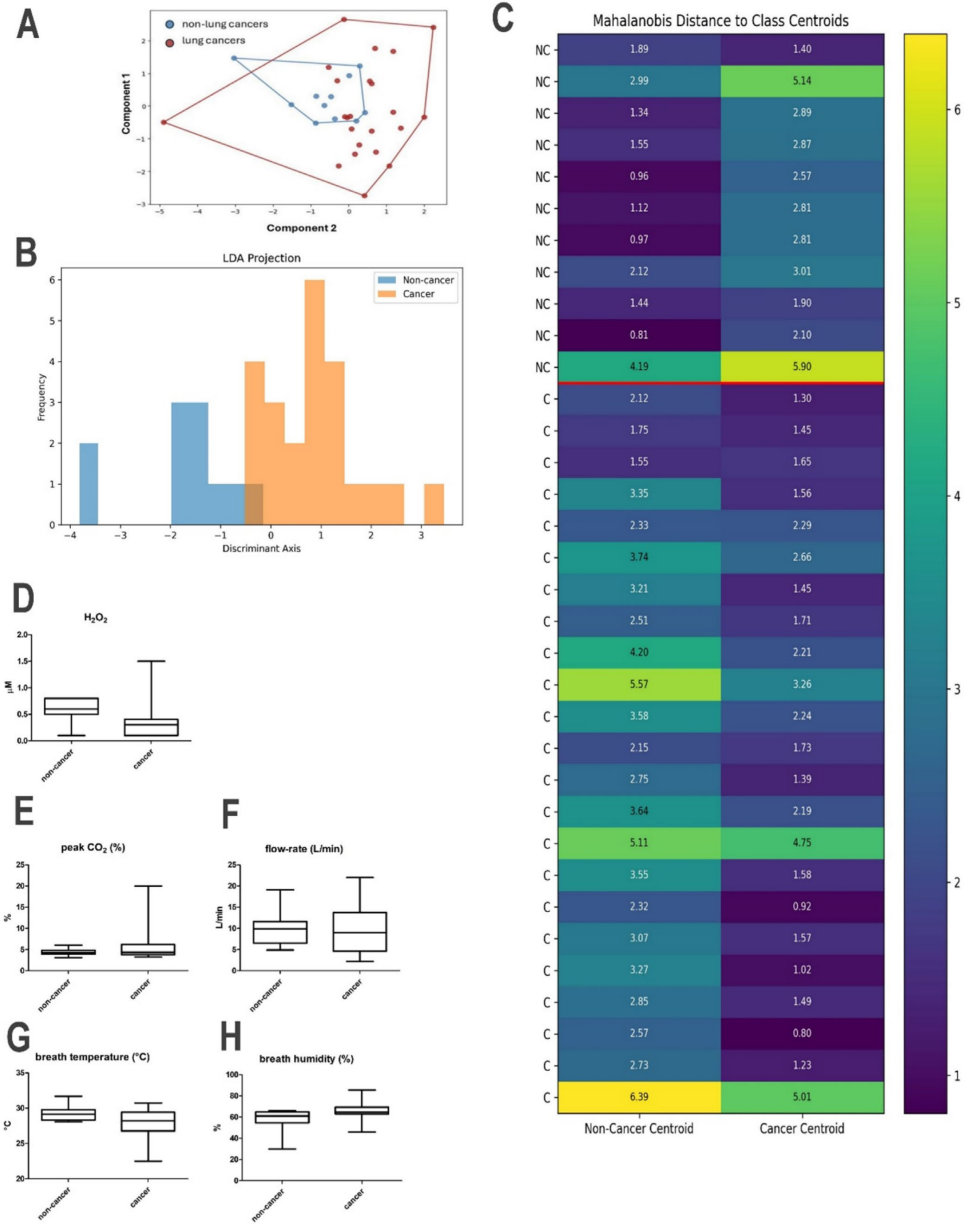
Multivariate separation of lung cancer and TLHC‐excluded participants in EBC feature space and univariate comparison of exhaled breath condensate (EBC)–derived physiological and biochemical variables between cancer and non‐cancer participants. (A) Principal component analysis (PCA) of standardized EBC variables showing projection onto the first two principal components. Convex hulls depict within‐group dispersion. (B) Linear discriminant analysis (LDA) projection onto the single discriminant axis maximizing between‐class separation. (C) Heatmap of Mahalanobis distances from each participant to cancer and non‐cancer class centroids using pooled covariance, illustrating covariance‐adjusted multivariate proximity. (D–H) Box‐and‐whisker plots summarizing individual EBC‐derived variables for non‐cancer and cancer participants: (D) hydrogen peroxide (H_2_O_2_), (E) peak end‐tidal CO_2_ (%), (F) exhalation flow rate (L/min), (G) breath temperature (°C), and (H) breath humidity (%). Boxes represent the interquartile range (IQR; 25th–75th percentiles), the central line denotes the median, and whiskers indicate the minimum and maximum observed values.

Unsupervised principal component analysis (PCA) of standardized exhaled breath condensate (EBC) variables demonstrated partial but discernible separation between patients with confirmed lung cancer and participants excluded from further investigation within the Greater Manchester Targeted Lung Health Check (TLHC) programme (Figure 2A). Projection onto the first two principal components (PC1 and PC2), which capture the greatest proportion of overall variance in the dataset, revealed distinct geometric structuring of the two groups within multivariate space.

Cancer cases exhibited greater dispersion, occupying a broader region of principal component space, whereas TLHC‐excluded participants were comparatively more compactly clustered. The convex hulls drawn around each group illustrate this difference in within‐group variance and visually demonstrate that the groups occupy partially distinct regions of multivariate space despite projection‐induced overlap. We emphasize that a visual overlap in a two‐dimensional PCA projection does not preclude separation in the full multivariate space, as PCA maximizes total variance rather than class discrimination and may not preserve class‐separating directions. This is particularly relevant in biological datasets where class differences may be distributed across multiple correlated variables rather than along a single dominant axis. Importantly, PCA is an unsupervised transformation that maximizes total variance without knowledge of class labels; therefore, any visible separation observed in PCA space reflects a true intrinsic (mathematically governed) multivariate difference rather than an artificial model‐driven discrimination.

To formally test whether a linear axis exists that maximally separates the two groups, linear discriminant analysis (LDA) was performed (Figure 2B). Unlike PCA, LDA is a supervised projection that identifies the axis maximizing the ratio of between‐class variance to within‐class variance. In this binary setting, LDA yields a single discriminant axis. Projection onto this axis demonstrated clear directional separation between cancer and non‐cancer participants, indicating the presence of an intrinsic linear combination of EBC features along which group centroids are maximally distinct. The observed separation is therefore not merely a function of variance structure but reflects, again, an intrinsic property of the two groups with a mathematically defined discriminative axis.

To further quantify multivariate separation while accounting for covariance structure, Mahalanobis distances [12], were computed from each individual data‐point to both the cancer and non‐cancer class centroids (Figure 2C). Mahalanobis distance incorporates feature correlations and scaling, providing a covariance‐adjusted metric of geometric proximity in high‐dimensional space. Unlike Euclidean distance, Mahalanobis distance reflects how atypical a data point is relative to the underlying covariance structure, thereby capturing deviations that may be masked in low‐dimensional projections. The resulting heatmap demonstrates that, in the majority of cases, individuals are closer (in Mahalanobis space) to the centroid of their true class than to that of the opposing class. Although the absolute distances are modest, the consistency of this pattern across participants indicates structured multivariate organization rather than random dispersion. Such patterns are often subtle and spread across several related measurements, so they may not appear as clearly separated groups on simple 2D plots. In clinical datasets, this is common, as disease‐related changes are rarely driven by a single variable but instead reflect small shifts across multiple physiological parameters. Multivariate models can combine these small differences across variables, allowing them to detect clinically meaningful patterns that are not obvious on visual inspection alone.

This pattern indicates coherent class‐specific clustering and supports the presence of an intrinsic multivariate separation between the cancer and control participants in the ExPeL study. Collectively, the PCA geometry, LDA discriminant axis, and covariance‐adjusted distance mapping converge on a consistent finding: cancer and TLHC‐excluded participants occupy distinguishable regions of EBC feature space.

Although TLHC‐excluded participants were not defined by long‐term benign follow‐up but rather by screening exclusion criteria under BTS and NICE guidance [13], the observed multivariate separation suggests that the EBC signal reflects intrinsic physiological differences between confirmed malignancy and screening‐negative individuals. These findings support the biological plausibility of EBC‐derived features as discriminative biomarkers in this feasibility cohort.

### 3.3 Distribution of Individual EBC and Breath Physiology Variables

Comparisons of individual EBC‐derived variables revealed differences in distributional patterns between cancer and non‐cancer participants (Figure 2B–F). Non‐cancer participants showed higher median hydrogen peroxide concentrations, while cancer cases exhibited lower central tendency with greater dispersion, including a high outlier. Peak end‐tidal CO_2_ values displayed a wider range and higher upper extremes among cancer cases. Exhalation flow rate varied substantially in cancer participants, spanning both low and high values, whereas non‐cancer values were more constrained. Breath temperature in cancer cases was modestly lower on average and more variable, and humidity showed a higher median and broader spread relative to controls.

### 3.4 Performance of Ensemble Models for EBC‐Based Classification

EBC features were used to train supervised machine‐learning models to classify and predict cancer versus non‐cancer status. The Voting Ensemble Model outperformed the Stacking Ensemble Model across multiple metrics (Table 2). The voting model achieved an accuracy of 85.7%, with perfect specificity (1.000) and precision (1.000), indicating strong discrimination of controls and minimal false positives. Its Matthews correlation coefficient (MCC) was 0.73, with a ROC–AUC of 0.90.

**TABLE 2:** Performance metrics for ensemble models predicting lung health outcomes from EBC profiles. Comparison of the classification performance of two ensemble models—a Voting Ensemble and a Stacking Ensemble, on test data derived from SMOTED EBC features, distinguishing lung pathology (cases) vs. control status. Metrics include standard binary classification indices: Accuracy, sensitivity, specificity, predictive values (PPV, NPV), error rates, MCC as a balanced correlation measure under class imbalance, and ROC‐AUC for discriminatory ability.

Metric	Voting ensemble model	Stacking ensemble model
Accuracy (%)	85.71	71.43
Sensitivity/Recall	0.800	0.800
Specificity	1.000	0.500
Precision (PPV)	1.000	0.800
Negative predictive value (NPV)	0.667	0.500
False positive rate (FPR)	0.000	0.500
Matthews correlation coefficient (MCC)	0.730	0.300
ROC‐AUC score	0.900	0.900
Misclassification rate (%)	14.29	28.57
Confusion matrix (TN, FP; FN, TP)	(2, 0; 1, 4)	(1, 1; 1, 4)

In contrast, the stacking ensemble achieved an accuracy of 71.4%, lower specificity (0.50), and an MCC of 0.30, while maintaining similar sensitivity and ROC–AUC. These results demonstrate the superior overall performance and stability of the voting ensemble in this dataset.

### 3.5 Untargeted LC–MS Metabolomic Profiling of EBC

**FIGURE 3:**
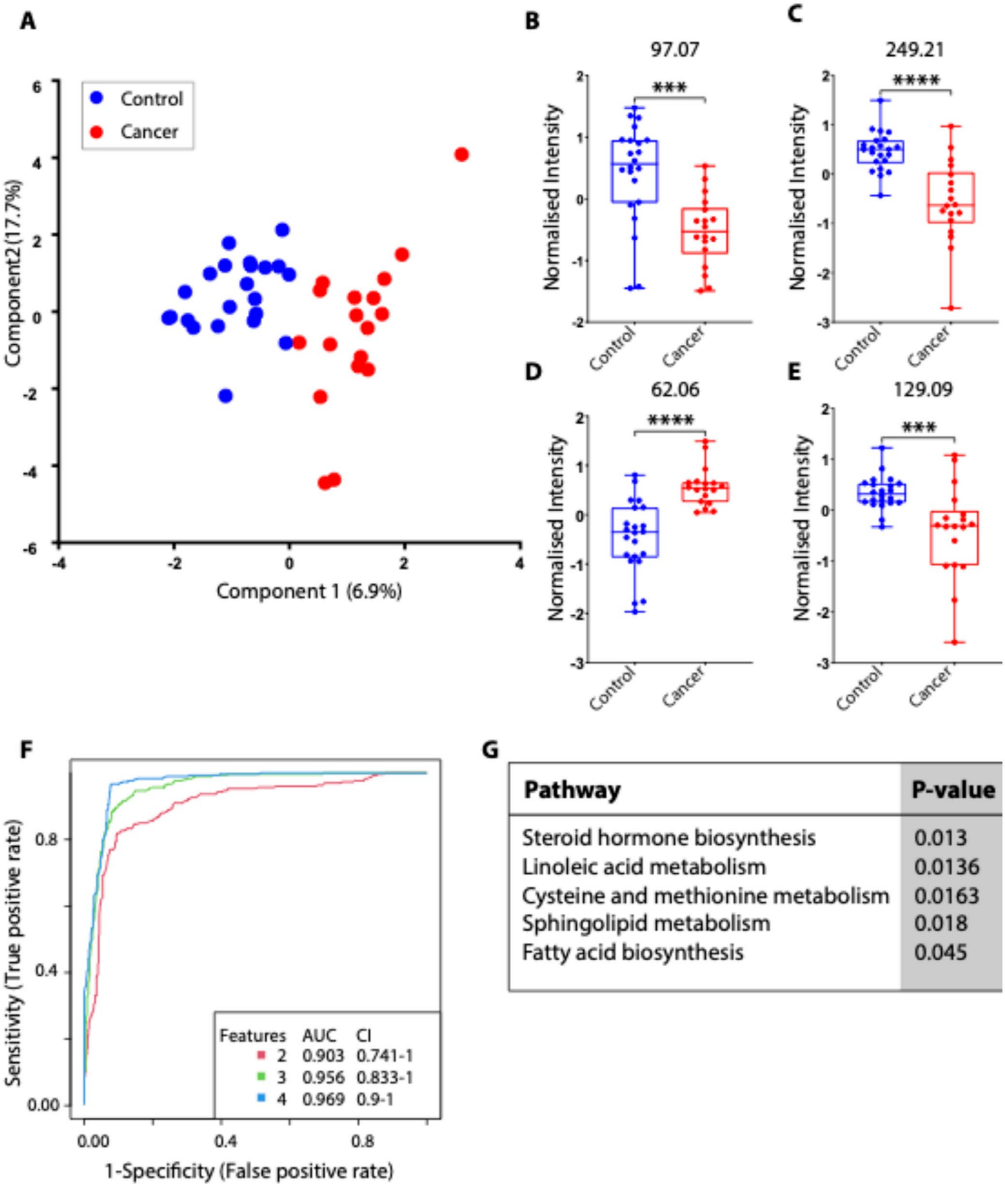
Exhaled breath condensate exploratory analysis. (A) sPLS‐DA scores plot using two components and 10 features per component. Average error rate of classification was 15.9%, in classifying non‐cancer controls (blue dots) against lung cancers (red dots) participants. (B–E) show box plots comparing means of four *m*/*z* values 97.97, 249.21, 62.06, and 129.09, which were top four features selected based on VIP scores (VIP > 1) and *t*‐test (*p* < 0.001) combined. F Multivariate receiver operating characteristic (mROC) curve using PLS‐DA and combination of 2, 3, and 4 features. The AUC was observed to be between 0.95 and 0.99 when incrementally changing from 2 to 4 features to perform the analysis. G Functional pathway analysis performed using mummichog algorithm [11], with a *p*‐value cut‐off threshold of 0.25. The percentages adjacent to the latent components indicate the proportion of variance in the predictor matrix captured by each component and are not intended as a measure of overall predictive performance.

Untargeted liquid chromatography–mass spectrometry (LC–MS) analysis of EBC identified 2132 molecular features across samples. Sparse partial least squares discriminant analysis (sPLS‐DA) was used as a supervised classification method and achieved 84.1% classification accuracy (Figure 3A). The percentages shown for the latent components reflect the proportion of variance in the predictor matrix captured by each component and should not be interpreted as a direct measure of discriminatory performance. Features were ranked using variable importance in projection (VIP) scores, and the top four discriminative molecules were selected for further analysis (Figure 3B–E).

Three of the four features were identified with high confidence. Tri(propylene glycol) butyl ether was annotated at Metabolomics Standards Initiative (MSI) level 3 based on spectral library matching. Two additional features were assigned molecular formulas C_7_H_10_O (putatively Hept‐4‐yn‐2‐one) and C_2_H_6_NO, although definitive structural annotation was not possible. These four metabolites differentiated cancer from control samples with an AUC of 0.969 (Figure 3F). Smoking status did not confound this analysis.

Network‐based pathway analysis revealed enrichment of metabolic pathways including sphingolipid and fatty acid biosynthesis (Figure 3G). These metabolites represent volatile or semi‐volatile compounds detectable in EBC during tidal breathing, highlighting the capacity of LC–MS profiling to capture biochemical signatures present in exhaled breath.

## 4 Discussion

The ExPeL study demonstrates that it was possible to distinguish early‐stage lung cancers from at‐risk controls via machine learning algorithms that were trained on exhaled breath condensate (EBC) data. EBC analyses is a non‐invasive, tidal‐breathing based measurement, and for this study, we used the Inflammacheck device. By integrating physiological breath features (oxidative stress, gas exchange, airflow, temperature, and humidity) with molecular profiling of volatile organic compounds (VOCs) captured during normal tidal breathing, this pilot study highlights a rapid, low‐cost, safe, and scalable adjunct to low‐dose CT (LDCT) screening within the UK's Targeted Lung Health Check (TLHC) programme. The overarching aim of this study is to support early detection and prioritization for imaging, improving efficiency in resource‐intensive national screening programmes.

### 4.1 Relevance of Control Definition and Real‐World Screening Context

Control participants were defined as ever‐smokers undergoing TLHC‐based CT assessment who were not referred for further diagnostic escalation, consistent with NICE and British Thoracic Society guidance for low‐risk pulmonary nodules [13]. This risk‐based classification provides a biologically and clinically relevant comparator, reflecting the population that would realistically be triaged in a screening context. Consequently, the differences in EBC features observed in this study correlate to early pathological variations rather than overt, advanced disease, enhancing the early‐stage translational validity of our findings.

### 4.2 Physiological EBC Signatures

Analysis of EBC variables revealed increased dispersion in hydrogen peroxide, end‐tidal CO_2_, exhalation flow, temperature, and humidity among cancer cases, consistent with clinically observed symptoms such as cough, wheeze, and exertional dyspnoea, secondary to localized airway oxidative stress, reduced exercise tolerance due to inefficient ventilation–perfusion matching, and inflammatory tone in early malignancy. These subtle physiological variations are difficult to detect using conventional univariate methods, highlighting the importance of supervised machine‐learning models to process and compute multivariate patterns.

When considering exhaled breath condensate H_2_O_2_ levels in early‐stage, newly diagnosed cancer patients versus controls, we acknowledge the powerful influence of confounding factors. Newly diagnosed lung‐cancer patients who might be clinically symptomatic following spirometry and FeNO assessments would normally be prescribed steroid inhalers. The steroid in these inhalers potentially might downregulate the production of NF‐κB [14, 15]. By suppressing NF‐κB, steroid inhalers could reduce the transcription of many inflammatory genes, leading to an overall dampening of the inflammatory response in the airways. Since H_2_O_2_ in EBC is primarily considered a marker of oxidative stress and inflammation in the airways [16, 17], the anti‐inflammatory effects of steroid inhalers directly lead to a reduction in EBC H_2_O_2_ levels [18].

The contrasting observations between the early‐stage disease in this ExPeL study and more advanced lung‐cancer cohorts such as noted in the VICTORY study [8], may reflect stage‐dependent shifts in airway oxidative biology. While early malignancy may exhibit subtle or confounded oxidative signatures, advanced lung cancer is associated with a strongly pro‐oxidative microenvironment in which tumor‐driven reactive oxygen species production [19], outweighs any inhibitory influences, resulting in higher exhaled H_2_O_2_ levels [6].

### 4.3 Machine Learning Insights

The Voting Ensemble Model, trained on SMOTE‐balanced EBC data to address class imbalance in a small sample, achieved > 85% accuracy, perfect specificity (1.0), high precision (1.0), and robust balanced performance (MCC 0.73, ROC‐AUC 0.90). Importantly, the accuracy metric should not be misinterpreted as approximately 20% of cancers being “missed”, as these results reflect model performance within a low‐prevalence, hold‐out test set; in real‐world TLHC populations, where cancer prevalence is low (~1%–2% per year among screened ever‐smokers in Greater Manchester), predictive values must be interpreted in context. High specificity and precision are particularly valuable in screening, as they minimize unnecessary downstream imaging and reduce patient anxiety and resource burden.

### 4.4 Molecular VOC Signatures From LC–MS Metabolomics

Untargeted LC–MS analysis of EBC identified over 2000 features, from which four key VOCs were able to discriminate cancer from control samples with an AUC of 0.969. These metabolites, detected during normal tidal breathing, provide a glimpse into the biochemical microenvironment of the respiratory tract.

We have also identified individual molecules that are associated with lung cancer, which will be added to inflammacheck in the future to improve performance. Two of the top four differentially expressed molecules could be identified. Tri(propylene glycol) butyl ether is an organic solvent that is used in multiple cleaning products, meaning that most people are likely to be regularly exposed [20]. It is not known to be produced as a product of intrinsic metabolism; hence it is most likely that one of the enzymes involved in its degradation pathway has been disrupted by cancer leading to overrepresentation in the cases in this study. C_7_H_10_O was identified as Hept‐4YN‐2‐one, for which there is little in the literature. Related molecules have been detected as part of a discriminator in the urine of patients with lung cancer previously [21].

The pathway analysis on all differentially expressed molecules provided insight into alterations in the respiratory tract in early‐stage lung cancer. These included up‐regulation of systems well characterized in cancer such as sphingolipids [22] and lipid metabolism [23]. The other pathways identified are less established, but there are studies supporting their roles in lung cancer. For example, linoleic acid expression in platelets has previously been suggested as a biomarker of non‐small cell lung cancer [24] and steroid biosynthesis has been demonstrated in lung cancer cell lines, which may have a role in immunomodulation [25].

### 4.5 Clinical Implications and Future Directions

Although a proportion of TLHC‐excluded participants had small or low‐risk pulmonary nodules that did not meet criteria for further escalation, classification within the present study reflects their clinical status at the point of discharge under BTS‐guided screening pathways. Thus, the observed discrimination between cancer and non‐cancer groups pertains to contemporaneous clinical decision‐making rather than retrospective confirmation of long‐term benignity. In this context, the identification of intrinsic multivariate separation supports the potential utility of an adjunct physiological assay, such as Inflammacheck, to provide an additional layer of biological risk stratification alongside imaging. This may be particularly valuable not only within structured screening programmes but also in primary care settings, where general practitioners are frequently required to decide whether to refer patients presenting with non‐specific yet concerning symptoms—such as persistent cough, unexplained weight loss, or haemoptysis that often overlap with benign respiratory conditions. An accessible adjunct test capable of reflecting underlying biological perturbation could therefore help reduce diagnostic uncertainty in this gray clinical zone, supporting more informed referral decisions while avoiding unnecessary escalation in low‐risk presentations.

We propose the feasibility of integrating EBC‐based physiological and VOC signatures into a predictive model in future; the ExPeL study provides a proof‐of‐concept for a non‐invasive triage tool upstream of LDCT.

Such an approach could prioritize high‐risk individuals for imaging, improving the yield of positive diagnoses per scan. It may also reduce radiation exposure and healthcare resource burden, particularly in low‐prevalence screening populations. The results of this study may enable mechanistically informed biomarker selection, leading to improvements of the Inflammacheck platform.

Future work will require prospective validation in larger multi‐centre trials employing TLHC populations, evaluating performance across diverse patient subgroups and exploring integration of newly identified VOCs into the predictive algorithm. By combining **machine** learning, multivariate physiological signals, and VOC metabolomics, EBC‐based approaches hold promise as scalable, rapid, and clinically actionable tools to enhance early lung cancer detection.

### 4.6 Limitations of the Study

A key limitation of this study relates to the definition of the control group. Controls were derived from screen‐negative, low‐risk participants within the Greater Manchester Targeted Lung Health Check programme rather than individuals with confirmed long‐term benign status. While some participants had small or low‐risk pulmonary nodules classified according to established clinical guidance, discharge from the screening pathway reflects real‐world decision‐making in contemporary CT‐based lung cancer screening programmes. Therefore, our control cohort represents a pragmatic screening population rather than a lifelong benign comparator, which should be considered when interpreting diagnostic performance estimates. As a pilot study, the sample size is small and findings will require validation in a larger cohort.

## Author Contributions

L.D., D.K.T., and S.K. wrote the manuscript. D.K.T., S.K., W.A., A.C., S.G., and N.D.B. designed the research. D.K.P. and L.D. performed the research and analyzed the data.

## Funding

This study was sponsored by Exhalation Technology Ltd. through in‐kind contributions of test devices and kits and the Northern Care Alliance Foundation Trust and funded by the Wellcome Trust (Grant code: R126087).

## Conflicts of Interest

D.P., L.D., W.A., S.G., D.K.T., S.K. report no conflicts of interest. N.B. has received payments for speaking from Chiesi, Sanofi and Glaxosmithkline (GSK), support for attending meetings from Astrazeneca (AZ), Chiesi and Sanofi and participates on an advisory board for Sanofi, Chiesi and TEVA. A.C. is Chief Respiratory Officer of University of Portsmouth Hospital NHS Trust Board.

## Supporting information


**Supporting Information: S1.** Detailed definition of control cohort.
